# Gifted but Misunderstood? An Interpretive Systematic Review of Gifted Education Policy, Practice, and Socio-Emotional Experience in England

**DOI:** 10.3390/jintelligence14030034

**Published:** 2026-02-24

**Authors:** Simge Karakaş Mısır, Michael Thomas

**Affiliations:** 1 Faculty of Education, Department of Special Education, Tokat Gaziosmanpaşa University, Tokat 60150, Türkiye; 2 Liverpool Institute for Research in Education (LIFE), School of Education, Liverpool John Moores University, Liverpool L1 9DE, UK

**Keywords:** England, gifted education, policy implementation, socio-emotional outcome, systematic mapping

## Abstract

This systematic review analyses the evolution of gifted education in England between 2010 and 2025. The year 2010 serves as a critical turning point, characterized by the withdrawal of the national Gifted and Talented (G&T) policy and the subsequent delegation of identification and provision responsibilities to schools. This change created a gap in the literature due to a lack of focused research examining the challenges and deficiencies that emerged following this policy shift. This study is original in that it is the first to bridge existing implementation gaps and provide a robust evidence base for future educational policies. The review focuses on policy frameworks, identification models, and socio-emotional outcomes. Following the PRISMA guidelines, fifteen peer-reviewed studies retrieved from Web of Science, Scopus, and Google Scholar were examined through thematic synthesis. Findings indicate a persistent gap between policy rhetoric and classroom practice. Identification processes remain heavily reliant on standardized testing and teacher judgment, often neglecting creativity, diversity, and contextual factors. Fragmented teacher training limits the ability to effectively support gifted learners, particularly those from disadvantaged or twice exceptional (2e) backgrounds. Socio-emotional outcomes reveal that academic success does not guarantee emotional well-being, highlighting the prevalence of perfectionism and stigmatization. These findings underscore the need for teachers and teacher educators to strengthen pre- and in-service training, so they can better recognize diverse forms of giftedness and support students’ socio-emotional needs through more equitable and research-informed practices.

## 1. Introduction

While the education of gifted students has been a constantly changing and debated area in international education (Tourón & Freeman, 2018), there is no single, universally accepted definition of giftedness (Subotnik et al., 2011). Historically, the concept of giftedness has been rooted in psychometric approaches, and early studies emphasized the measurement of Intelligence Quotient (IQ) as the primary indicator of giftedness (Carman, 2013). However, contemporary research has shifted towards a more dynamic and multidimensional understanding of giftedness and acknowledged the interaction of genetic, environmental and sociocultural factors in the development of exceptional abilities (Heller, 2004). This paradigm shift has important implications for educational practice, particularly in promoting inclusive and equitable learning environments for gifted students.

Educational policies play a pivotal role in the identification of and provision for gifted students, a field that has evolved in England over decades under the influence of national and international frameworks (Koshy et al., 2010a). In this context, the Warnock Report (1978) marked a significant milestone by recognizing giftedness within special educational needs and emphasizing that 15–20% of the student population might require specialized support during their schooling (Eyre, 2003/2016). Despite the early recognition provided by the Warnock Report, gifted education in England has witnessed a dynamic process of transformation driven by societal expectations and scientific advancements. Although the UK Government has aimed to assist these students in maximizing their potential, this process has not been immune to challenges and criticism. Practices that were limited to civil society and individual school initiatives from the 1970s onwards gained the status of centralized policy in the late 1990s with the ‘Excellence in Cities’ (EiC) initiative and the national ‘Gifted and Talented’ (G&T) programme. While attempts were made to integrate pedagogical innovations such as multiple intelligences theory and teacher training into the system during this period, these centralized initiatives became the focus of structural criticism regarding consistency and sustainability. In 2010–2011, the UK Government formally discontinued the Gifted and Talented Programme due to concerns regarding its effectiveness and the lack of specialist support. Nevertheless, schools remained responsible for supporting their most able students. Today, despite the absence of a national policy, schools continue to implement various initiatives to meet these educational needs. Although empirical research on gifted education in England has increased in recent years, there remains a notable absence of systematic reviews that comprehensively examine how the field has evolved following the discontinuation of the national Gifted and Talented (G&T) program in 2010. The year 2010 marks a significant structural shift in which central policy mandates were withdrawn and responsibility for gifted provision was transferred to local school initiatives, making it an analytically meaningful starting point. Accordingly, the present study aims to address this longstanding gap in the literature by systematically mapping how schools interpreted, implemented, and developed gifted education in the absence of a national framework during the period 2010–2025. Accordingly, this study aims to address these questions by examining research to provide a comprehensive overview of the field, identify emerging trends and offer recommendations for future research and policy development.

## 2. Conceptualizing Giftedness in England

### 2.1. Definitions and Theoretical Frameworks

The concept of giftedness occupies a central place in extensive academic debates, shaped by numerous definitions and theoretical models. Historically, giftedness has often been defined by high Intelligence Quotient (IQ) scores representing approximately the top 2–3% of the population; this approach was rooted in the work of psychologists such as Binet and Simon (1948) and Terman (1916). However, contemporary perspectives acknowledge that giftedness is not limited to IQ alone but encompasses a broader set of abilities and has a multidimensional structure (Carman, 2013; Worrell et al., 2019). Renzulli’s (2021) Three-Ring Conception of Giftedness defines gifted behavior as the interaction of three factors—above average cognitive ability, strong task commitment, and creativity—emphasizing that giftedness is not static but context-dependent and responsive to developmental processes. Gagné’s (2018) Differentiated Model of Giftedness and Talent (DMGT) distinguishes between “giftedness” as natural abilities and “talent” as systematically developed skills, highlighting the critical role of environmental and intrapersonal catalysts in transforming potential into talent.

Psychometric and cognitive theories, such as Sternberg’s (2020) Theory of Successful Intelligence, integrate analytical, creative, and practical intelligence, frame giftedness in terms of adaptability and problem solving skills in real life contexts. Gardner’s (2011) Theory of Multiple Intelligences identifies eight different types of intelligence, such as linguistic, logical-mathematical, and musical and asserts that giftedness can emerge in a wide range of areas beyond academic domains.

Among developmental and systems approaches, Dabrowski’s (1964) Theory of Positive Disintegration focuses on the emotional and ethical development of gifted individuals, arguing that advancement in their areas of interest is directly linked to intrinsic motivation (Mendaglio, 2012). Subotnik et al.’s (2019) Gifted Development Model views giftedness as a dynamic process evolving from potential to expertise, shaped by both individual characteristics and environmental conditions. One of the leading talent development models, Renzulli’s Schoolwide Enrichment Model (SEM), aims to provide enrichment opportunities to all students while offering creativity-driven, engagement-focused special programs for gifted learners (Reis et al., 2021). Stanley’s Talent Search Model identifies academically gifted students through above-grade-level testing and offers them accelerated educational opportunities (Assouline & Foley-Nicpon, 2021).

From a contemporary perspective, giftedness is not a fixed attribute but rather a multidimensional and evolving construct shaped by the interaction of cognitive potential, creativity, motivation, and contextual factors (Pfeiffer, 2023; Subotnik et al., 2019). While early definitions focused primarily on intellectual capacity, modern models acknowledge that giftedness can manifest in diverse ways and emphasize the critical role of talent development, social emotional wellbeing, and environmental influences in supporting exceptional abilities. This multidimensional understanding aligns with current approaches in England, which prioritize school-based, individualized support strategies over a centralized national policy.

### 2.2. Educational Approaches for Gifted Students

Gifted students require tailored educational approaches to ensure their intellectual and personal development. In England, various strategies have been used to meet their needs, including enrichment, acceleration, differentiation within mainstream classrooms, and specialized programs. Each approach has its advantages and challenges, which in turn influence its implementation and effectiveness. (Freeman, 2013; Tomlinson, 2014)

One of the most widely recognized methods is enrichment, which enhances the standard curriculum by incorporating deeper and broader learning experiences. This can include independent research projects, mentorship programs, and participation in extracurricular activities that foster creativity and critical thinking (VanTassel-Baska, 2003). Enrichment allows gifted students to explore subjects in greater depth without moving beyond their designated grade level, providing intellectual stimulation while maintaining social integration with peers.

Alternatively, acceleration enables gifted students to progress through the educational system at a faster pace. This can take several forms, including grade skipping, subject-specific acceleration, and early entry to university (Colangelo et al., 2012). Research suggests that acceleration is highly effective in promoting academic achievement and motivation among gifted students (Bernstein et al., 2021). However, concerns about emotional and social development sometimes deter schools from adopting this approach (Rimm et al., 2018). A widely used approach in England is differentiation within mainstream classrooms, where teachers adapt instruction to accommodate varying ability levels. This method includes flexible grouping, tiered assignments, and inquiry-based learning (Tomlinson, 2001). While differentiation promotes inclusive education, its effectiveness largely depends on teacher expertise and institutional support. Studies indicate that many educators feel unprepared to effectively differentiate gifted students due to limited training in gifted education (Hodges et al., 2018).

In addition to these strategies, some gifted students benefit from specialized programs and schools. England has a small number of selective grammar schools and independent schools that offer advanced curricula tailored to high-achieving learners. Additionally, some universities provide early admission or extension programs for exceptionally gifted students (Freeman, 2010). These specialized provisions can offer highly challenging academic experiences but may raise concerns about equity and accessibility, as admission to selective institutions is often influenced by socioeconomic factors (Campbell et al., 2007).

Despite the availability of multiple educational approaches, significant challenges remain vis-à-vis their implementation. Many schools lack the necessary funding and resources to fully support gifted education program. Furthermore, teacher training in gifted education is often insufficient, leading to inconsistent practices across schools (Gross, 2006). Equity concerns also persist, as students from disadvantaged backgrounds may have less access to enrichment opportunities and selective programs (Rimm et al., 2018). Addressing these issues requires a stronger policy framework, increased professional development for educators, and greater investment in resources for gifted learners.

### 2.3. Historical Evolution of Gifted Education Policies in UK

In the context of the English education system, critics have argued that despite the emergence of the concept of giftedness, there has been no consistent national policy or systematic framework for the education of gifted students (Koshy et al., 2010b; Dimitriadis, 2012b). Instead of a single, unified policy or curriculum approach, the landscape has been characterized by localized and fragmented practices, often driven by school autonomy rather than central guidance. An examination of national changes in education policies suggests that programs have undergone significant fluctuations, particularly following the discontinuation of the national Gifted and Talented (G&T) initiative in 2010, which led to a shift from centralized mandates to diverse, ad hoc school-based approaches (Casey et al., 2011; Dimitriadis, 2016).

In England, the education of gifted students relies on several key policies that have been influential in shaping provision. The Warnock Report (1978), published in England for special education, marked an important moment in the recognition of special educational needs, including gifted students. The report emphasized the importance of special education interventions and emphasized that 15–20% of students may need special support at some point during their school life (Warnock Report, 1978). Despite this early recognition, the implementation of the gifted education program in England has been inconsistent and often affected by wider educational reforms and resource constraints (Freeman, 2005). The distinction between ‘gifted’ and ‘talented’ became more clearly defined in the 1990s and 2000s. Policies such as the Excellence in Cities (EiC) initiative were introduced to identify and support gifted and talented (G&T) students in schools. This period marked a shift towards formalising the identification and provision of gifted students. It required schools to register these students and provide appropriate educational opportunities for them. However, in 2010–2011 the UK Government formally terminated the G&T programme. This decision was driven by the lack of effectiveness and adequate specialist support for the program. Despite the cessation of the national program, schools retained their responsibility to support their most able students and ensured that gifted education continued at an institutional level (DCSF, 2008). This historical overview reflects the continuing challenges and changing priorities that influence the needs of gifted students in the English education system.

## 3. Methodology

### 3.1. Research Question and Approach to Review

This study employed a systematic review method using the updated 2020 PRISMA (Preferred Reporting Items for Systematic Reviews and Meta-Analyses) guidelines (Page et al., 2021). The review was conducted in accordance with PRISMA guidelines (see Supplementary Material). Furthermore, the Systematic Mapping (SM) methodology was utilized as it provided detailed evaluation criteria to enable an in-depth examination of topics (Dicheva et al., 2015). This methodology offered a structured framework for categorizing published reports and their associated findings (Bond et al., 2020; Yusuf et al., 2024). The information derived from the application of SM was an effective preliminary step preceding subsequent research efforts and more detailed systematic reviews (Kitchenham et al., 2011). The study protocol was registered with the Open Science Framework (OSF) under the DOI 10.17605/OSF.IO/S2Z49. To conduct a comprehensive literature search, Web of Science, Scopus, and Google Scholar were selected as databases and the literature search spanned the years 2010 to 2025. Searches were last updated in November 2025. The review was structured around the following research questions:

RQ1. What is the distribution of studies conducted for the education of gifted students?

RQ2. What is the core thematic foci of academic studies in the field of gifted education?

RQ3. What are the fundamental models and frameworks used for the identification of gifted students?

RQ4. What are the reported academic and socio-emotional outcomes of gifted education interventions?

RQ5. What methodological patterns and quality characteristics can be observed in gifted education research?

**Figure 1:**
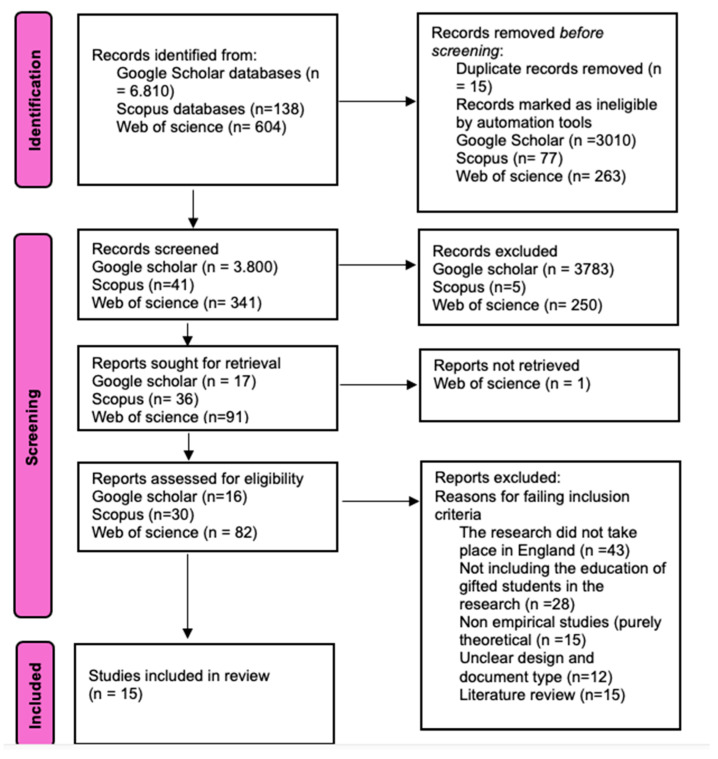
Inclusion/Exclusion Criteria and Screening Process.

The keywords listed in Table 1 were used as the initial search criteria for this study. The search terms yielded a total of 6810 publications in Google Scholar, 138 publications in Scopus, and 604 publications in Web of Science (see Figure 1).

In the PRISMA flow diagram, the term ‘records screened’ refers to the initial screening stage conducted following the removal of duplicates. During this process, the titles and abstracts of the remaining unique records were systematically evaluated based on the inclusion and exclusion criteria outlined in Table 2. Records that did not align with the scope of the study or failed to meet the established criteria were excluded at this stage; only those studies that passed this preliminary screening were advanced to the full-text eligibility assessment. The initial search string only utilized the established keywords. The subsequent step involved the individual examination of articles for their relevance to the research questions. The abstract of each article was assessed for its alignment with the study’s scope, and the papers were then refined using the criteria detailed in Figure 1.

Following the refinement of the initial search results based on the criteria outlined in Table 2, the articles were evaluated through full-text screening. This phase involved eight distinct focus points: timeframe, language, publication type, geographical focus, discipline areas, accessibility, methodology, and content focus. To further clarify the inclusion of articles, three essential questions were developed and used to examine the content:

- Do the articles concern the education of gifted students?
- Do the articles exclusively focus on the education of gifted students in England?
- Do the articles report an intervention or empirical study?

Studies to be included in the research were manually selected by the researchers, considering not only the predetermined inclusion and exclusion criteria but also their alignment with the defined characteristics. In this process, studies that only offered a theoretical framework or functioned as comprehensive literature reviews were excluded. As the goal was to reach the broadest possible range of applications and the focus of this work was the mapping of the research area, rather than a holistic synthesis of findings, publications were not filtered based on research quality. The 15 publications that met the inclusion criteria fell under the category of scholarly work in the field of teaching and learning, reflecting Kreber and Cranton’s (2000) description of research that integrates practitioner insights, instructional practices, and learner experiences.

### 3.2. Data Extraction

The year 2010 was adopted as the reference point, marking the formal discontinuation of the national Gifted and Talented (G&T) programme and the start of the transition from centralized policy to localized school autonomy; developments in gifted education in England from this date until 2025 were analysed through the included articles using systematic coding and thematic classification methodologies The coding process was performed manually. Articles were examined under the following subheadings to determine the focus areas in the identification and education of gifted individuals:

(a) publication year, (b) examined themes, (c) methodology, (d) study design, (e) concepts of giftedness examined, (f) tools and platforms used, and (g) reported educational outcomes. The process continued with a comprehensive full-text review of the studies manually selected based on their titles and abstracts after the initial mapping and classification. A total of 15 studies that met all the criteria were identified for final analysis.

### 3.3. Analysis

Data analysis combined descriptive statistics and qualitative content analysis to capture both the breadth and depth of the evidence base. Descriptive analysis was employed to illustrate the frequency and distribution of key variables, including (a) distribution of publication year, (b) focus area/subject domain, (c) identification framework/model, (d) academic/socio-emotional outcome, and (e) methodological pattern/quality. Frequencies and percentages were visualized through bar charts and tables to map the research landscape.

In addition to this quantitative overview, a narrative synthesis was conducted to interpret recurring patterns within these variables. This synthesis explored how different identification models were conceptualized, how policy frameworks were operationalized in schools, and how pedagogical strategies contributed to the academic, social, and emotional development of gifted students. Complementing this, content analysis was applied through a systematic manual coding process aimed at extracting recurrent themes regarding the challenges and opportunities of gifted education in England. This iterative process, involving multiple rounds of refinement and peer discussion, allowed for the identification of thematic clusters such as teacher capacity, policy and pedagogy, student voice, and equity of access. These themes provided a framework for linking empirical findings to broader debates on the evolution of gifted education policy and practice in the UK.

Table 3 presents the methodological characteristics of gifted education research in the UK. The methodological analysis of the core articles in this review reveals a diversity of research designs and data collection methods employed in the field. Descriptive analysis indicates that a substantial proportion of the included studies adopted a mixed-methods approach (Dimitriadis, 2016; Koshy et al., 2010b; Koshy & Pinheiro-Torres, 2013; Lamb & Aldous, 2014). This mixed approach primarily stems from the need to investigate policy implementations (Koshy et al., 2010b) and mentoring experiences (Lamb & Aldous, 2014) using both large-scale surveys and in-depth qualitative data.

Qualitative studies predominantly employed a case study design, focusing on gaining a deep understanding of the experiences of gifted students (Dimitriadis, 2012a, 2012b), their parents (Koshy et al., 2013), and teachers. Interviews (semi-structured and focus group discussions), observations, and document analysis were the primary methods for collecting qualitative data. Particularly in the examination of practices within mathematics education (Dimitriadis, 2012a, 2012b), case studies proved effective in investigating the effects of different organizational provisions on student attitudes and progress.

Quantitative methods, primarily through surveys utilizing Likert-type and open-ended questions, were generally used to measure teachers’ knowledge and confidence levels (Dimitriadis et al., 2021) or program coordinators’ perceptions of policy (Koshy et al., 2013; Koshy & Pinheiro-Torres, 2013) across a broader sample. The inclusion of cross-cultural (Cross et al., 2019) and disadvantaged group-focused studies (Koshy et al., 2013) enriches the field’s methodological scope, reflecting the effort of gifted education research to provide both breadth and depth.

## 4. Results

### 4.1. RQ1: What Is the Distribution of Studies Conducted for the Education of Gifted Students?

**Figure 2:**
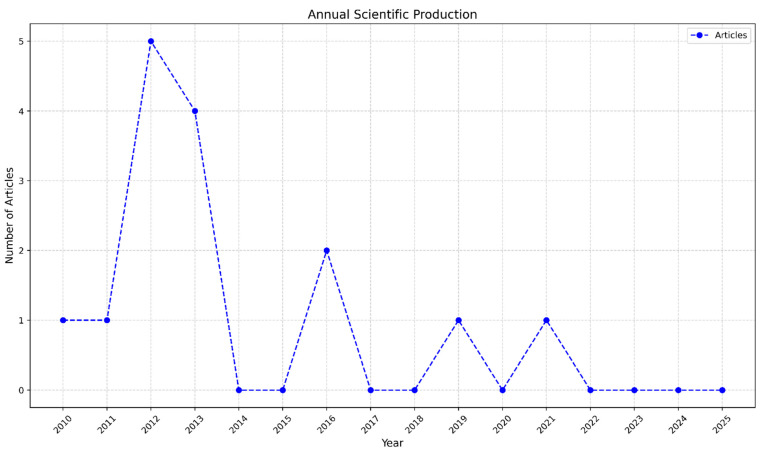
Annual scientific production in the decade 2010–2025.

The search results were acquired in bib format “BibTeX (.bib)”and processed using Bibliometrix software (Aria & Cuccurullo, 2017), which made it possible to extract basic information, publication details, and specific data from each article based on the initial categorization of the study. As a result of the systematic literature review, a total of 15 studies that met the criteria were found. Figure 2 shows the distribution of the studies by years across the period from 2010 to 2025.

Figure 2 illustrates the temporal distribution of the 15 identified studies focused on gifted education in England, showing that research output was not consistent over the specified period (2010–2025). A clear peak in production occurred in the year 2012 (*n* = 5 articles), followed by another significant output in 2013 (*n* = 4 articles). This intense research activity between 2012 and 2013 likely reflects the academic community’s response to or evaluation of the then-current Gifted and Talented (G&T) policy just before or around its discontinuation. Following this peak, a notable decline is observed, with zero production between 2014 and 2015, and subsequent years showing sporadic single or double publications (e.g., *n* = 2 in 2016). This fluctuating pattern suggests that while the field generated a concentrated body of work during the G&T policy’s final years, the overall research volume significantly decreased in the post-policy era, highlighting a diminished, yet intermittent, focus on the topic after 2013.

### 4.2. RQ2: What Are the Core Thematic Foci of Academic Studies in the Field of Gifted Education in England?

**Figure 3:**
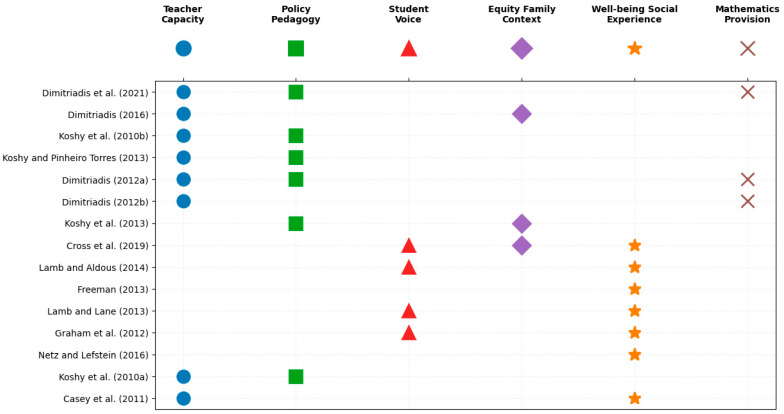
Thematic Mapping of Gifted Education Research in England (2010–2025) (Dimitriadis et al., 2021; Dimitriadis, 2016; Koshy et al., 2010b; Koshy & Pinheiro-Torres, 2013; Dimitriadis, 2012a, 2012b; Koshy et al., 2013; Cross et al., 2019; Lamb & Aldous, 2014; Freeman, 2013; Lamb & Lane, 2013; Graham et al., 2012; Netz & Lefstein, 2016; Koshy et al., 2010a; Casey et al., 2011).

In the initial phase of the analysis, a preliminary pool of codes was generated by extracting prominent concepts and recurring patterns from each publication. The subsequent synthesis of these codes, based on semantic affinities and theoretical interrelationships, revealed six dominant thematic areas (see Table 4 and see Figure 3). These studies reflect a systemic transformation in English gifted education since 2010, characterized by a shift from centralized policy to decentralized, school-based models. Among the identified themes, ‘teacher capacity’ emerges as a pivotal area of focus, highlighting its critical implications for the quality of educational practice.

Teacher capacity emerges as a pivotal determinant of educational quality. The literature indicates that teachers frequently lack the essential pedagogical knowledge to identify gifted students or design differentiated instruction (Dimitriadis, 2012a, 2012b; Koshy & Pinheiro-Torres, 2013). This deficit, compounded by insufficient professional development, compels a reliance on subjective judgment and leads to inconsistent standards across schools, directly undermining the effectiveness of national education policies.

Closely connected to teacher competence is the second theme, policy and pedagogy, which captures the evolving dynamics of England’s gifted education framework in the post–Gifted and Talented (G&T) era. A study by Koshy et al. (2010b) revealed that, following the discontinuation of the G&T program, educational governance shifted from centralized mandates to localized implementation. While this transition granted schools greater autonomy and flexibility, it also introduced fragmentation and inconsistency in practice. These findings underscore how policy ambiguity translates into uneven classroom practices, weakening the sustainability and equity of gifted education provision.

The implications of policy and pedagogical variation are particularly evident in the third theme, student voice, which foregrounds learners’ emotional and experiential perspectives. Research by Lamb and Aldous (2014) and Graham et al. (2012) demonstrates that students perceive being labelled as “gifted” as a double-edged experience on the one hand, fostering motivation and academic ambition, but on the other, generating social pressure and feelings of isolation. These findings highlight the crucial role of supportive teacher student relationships in sustaining self-efficacy and belonging among gifted learners. The emphasis on student voice reframes gifted education as not merely an academic process, but a complex psychosocial journey shaped by identity, recognition, and context.

Parallel to these learner-centred insights, the fourth theme, equity and family context, underscores the structural inequalities that shape access to gifted education. Koshy et al. (2013) found that students from lower socioeconomic backgrounds face significant barriers in accessing enrichment program and resources. Although parents’ express strong aspirations for their children’s success, limited financial means and insufficient guidance often restrict their ability to provide effective support. This body of research highlights the importance of conceptualizing gifted education not only as a cognitive or pedagogical concern but also as a matter of social justice and equal opportunity.

Complementing these perspectives, the fifth theme, well-being and social experience, focuses on the emotional challenges associated with giftedness. Conversely, mentoring and e-mentoring programs have been shown to strengthen students’ self-awareness, resilience, and social integration (Lamb & Aldous, 2014). These findings emphasize that gifted education must adopt a holistic approach that balances intellectual development with emotional health and interpersonal growth.

The sixth theme, mathematics provision, represents a distinctive disciplinary strand within English gifted education research. Dimitriadis (2012a, 2012b) explored various instructional models such as ability grouping, mentoring, and individual enrichment used to support mathematically gifted learners. His findings indicate that teacher expertise, manageable class sizes, and access to advanced learning opportunities are critical factors influencing student outcomes. This line of inquiry underscores the significance of subject-specific pedagogical approaches within the broader gifted education landscape.

These six themes cohesively demonstrate the evolution of gifted education in England from centralized standardization to localized innovation, where policy, pedagogy, and teacher expertise are intertwined. The studies suggest that the contemporary paradigm reflects an increasingly holistic and inclusive understanding that values not only academic excellence but also teacher professionalism, emotional balance, and equal opportunity.

### 4.3. RQ3: What Are the Fundamental Models and Frameworks Used for the Identification of Gifted Students in England?

The processes for the identification and classification of gifted students in England have historically been shaped by the interplay of national policy initiatives, school-based practices, and evolving theoretical models. The research examined indicates a continuous shift over time, moving from centralized and policy-driven approaches toward increasingly teacher-led and school-based identification systems (Koshy et al., 2010b; Dimitriadis, 2012a; Casey et al., 2011). This transformation has fostered a more holistic understanding that giftedness must be defined not merely through cognitive measures, but also through contextual, pedagogical, and socio-emotional dimensions.

#### 4.3.1. Policy-Based Frameworks and National Standards

The Gifted and Talented (G&T) Program, which was in effect from the late 1990s until 2011, constituted the cornerstone of central policy regarding the identification of gifted students in England. The Department for Education and Skills (DfES) mandated that schools identify approximately 5–10% of their student population as “gifted and talented” and maintain “register lists” for these students (DfES, 2003). The programme was conceptually supported by the Institutional Quality Standards (IQS) and Classroom Quality Standards (CQS), which were intended to ensure uniformity of practice across schools.

However, a large-scale review by Koshy et al. (2010b) revealed that these national standards were not consistently implemented on the ground. Many school coordinators were unaware of the standards, while others defined giftedness using a narrow metric based solely on test scores. This resulted in significant regional inequities and biases within the identification process. Furthermore, findings by Koshy and Pinheiro-Torres (2013) demonstrated that the policy’s primary focus on labelling students often relegated pedagogical development to a secondary concern, creating conceptual confusion among teachers. The eventual termination of the G&T program in 2011 shifted the identification process away from a centralized structure to school-based practices, a move that, while gaining flexibility, introduced new challenges concerning consistency and inclusivity (Koshy & Pinheiro-Torres, 2013).

#### 4.3.2. Teacher-Led and School-Based Identification Models

Following the abolition of the national program, the process for identifying gifted students increasingly relied on teacher observation and professional judgment. Dimitriadis (2012a, 2012b) found that in primary schools, teachers often identified mathematically gifted students not through IQ-based measures, but through observation, problem-solving performance, and classroom productivity. The four core models implemented by schools included:

- In-class differentiation
- Ability-based grouping (setting/levelling)
- Separate enrichment groups
- Mentoring programs

Among these models, mentoring and small group work emerged as the methods most strongly supporting academic achievement and motivation. This effect was particularly pronounced in provisions led by subject-specialist teachers (Dimitriadis, 2012b). Similarly, a four-year university-based intervention program evaluated by Casey et al. (2011) demonstrated increases in academic achievement, self-confidence, and aspirations for higher education among socioeconomically disadvantaged students. These findings indicate that long-term, multi-component models that include affective support create lasting impact for gifted students. However, variables such as teacher capacity, class size, and school resources continue to be determining factors in the quality of these implementations.

#### 4.3.3. Theoretical and Cognitive Approaches

Although policies and practices related to the identification of giftedness in England have evolved, numerous studies highlight a lack of theoretical coherence in the field. Dimitriadis (2016) emphasized that theoretical models such as Renzulli’s (1978) Three-Ring Model or Gagné’s (2004) Differentiated Model of Giftedness and Talent (DMGT) are applied in a limited manner during the identification process, thereby weakening the validity of gifted programs.

Theoretical frameworks are more visible within the domain of mathematics education. Teachers assess students’ cognitive depth using indicators of higher-order thinking, problem-solving, and creativity, often based on Bloom’s Taxonomy (Adams, 2015; Dimitriadis, 2012a). This approach conceptualizes giftedness as a dynamic process of performance and learning, differentiating it from static IQ measurements. However, research focusing on twice-exceptional (2e) students revealed that teachers are often inadequately prepared to identify both giftedness and learning difficulties concurrently (Dimitriadis et al., 2021). This finding suggests that the consistent application of robust theoretical models in identification remains limited.

#### 4.3.4. Contextual and Socio-Emotional Approaches

The reviewed literature has established that giftedness must be evaluated not only as a cognitive trait but also as a socio-emotional and environmental phenomenon. Research by Koshy et al. (2013) demonstrated that students from socioeconomically disadvantaged families often remain outside the formal identification system. Furthermore, Freeman’s (2013) longitudinal research indicated that the “gifted” label alone does not predict success; rather, lasting achievement is correlated with variables such as hard work, emotional support, and a positive outlook. Studies by Lamb and Aldous (2014) and Graham et al. (2012) found that students identified as gifted in areas like physical education and foreign languages encounter social exclusion, prejudice, and identity conflicts. These findings suggest that giftedness must be assessed alongside emotional, cultural, and social contexts, necessitating that identification models evolve into a structure that is inclusive, multi-dimensional, and culturally responsive.

The current literature on the identification of gifted students in England reveals that three primary models have emerged over time. Firstly, policy and standards-based models represent systems developed based on centralized regulations, prioritizing institutional accountability (Koshy et al., 2010b). Within this framework, schools were expected to identify a specific percentage of students as “gifted and talented” and create registration systems based on national quality standards. However, the effectiveness of these policies was limited due to issues such as lack of consistency at the implementation level, conceptual ambiguity among teachers, and an overemphasis on test-based criteria.

In contrast, teacher-led and school-based models adopted a flexible understanding of identification rooted in pedagogical differentiation and continuous teacher observations (Dimitriadis, 2012a, 2012b; Casey et al., 2011). This approach allowed students to be assessed using dynamic indicators such as creativity, problem-solving, and in-class performance. Mentoring and enrichment program implemented, particularly in disadvantaged areas, generated positive effects on students’ academic achievement and self-confidence. However, factors such as teachers’ professional competence, resource scarcity, and variability in implementation continue to emerge as limiting factors for the model’s sustainability.

Thirdly, theoretical and cognitive-based models offered a more in-depth identification perspective by associating giftedness not only with cognitive capacity but also with creativity, metacognitive awareness, and complex problem-solving skills (Dimitriadis, 2016). These models are grounded in established theoretical frameworks, such as Renzulli’s (1978) Three-Ring Model and Gagné’s (2004) Differentiated Model of Giftedness and Talent, utilizing dynamic performance indicators rather than static IQ measurements. The evolution of these three approaches demonstrates a transformation in the conceptualization of giftedness in England, moving from traditional psychometric criteria toward an ecological, pedagogical, and multi-dimensional understanding. Yet, the literature emphasizes that this transition is not yet complete, highlighting the critical need to establish theoretical coherence, mitigate socioeconomic inequalities, and enhance teachers’ competence in the identification processes. Therefore, future models for gifted identification must be placed within a more holistic, culturally responsive, and interdisciplinary framework.

Table 5 demonstrates a clear historical and conceptual evolution in England’s gifted identification landscape from policy-regulated frameworks toward school-centered, contextually responsive, and theoretically diverse approaches. The early centralized models (e.g., G&T Program) provided structural consistency but lacked inclusivity and adaptability. Following their discontinuation, teacher-led and theoretically grounded frameworks have enabled more nuanced, holistic identification processes.

### 4.4. RQ4: What Are the Reported Academic and Socio-Emotional Outcomes of Gifted Education Interventions?

Within the English context, interventions designed for gifted and talented students have been shown to influence not only academic performance but also affective, social, and identity development. The literature consistently indicates that the effectiveness of such interventions is closely linked to factors including program duration, multidimensional design, teacher expertise, and students’ socio-economic environments. For these programs to achieve long-term impact, they must support not only academic enrichment but also self-regulation, belonging, and self-efficacy (Casey et al., 2011; Dimitriadis, 2012a, 2012b).

Academic outcomes reveal that systematic and holistic interventions enhance cognitive advancement, motivation, and aspirations toward higher education. Long-term university–school partnerships implemented in disadvantaged areas have been shown to improve students’ academic performance as well as their confidence and educational ambition (Casey et al., 2011). Comparative research in mathematics education further demonstrates that the quality of organizational models significantly determines achievement levels. Dimitriadis (2012a, 2012b) emphasized that small-group and mentorship-based models yield the most substantial gains compared to classroom differentiation or ability grouping. When conducted under the guidance of subject specialists, such models foster deep conceptual understanding, strengthen problem-solving abilities, and enhance intrinsic motivation for learning. However, sustaining these gains requires theoretically grounded and continuously monitored programs; otherwise, the initial academic progress tends to diminish over time (Dimitriadis, 2016). Field specific differences also influence outcomes subjects like physical education and modern languages often rely heavily on teacher initiative, resulting in variability in practice and success rates (Lamb & Aldous, 2014; Graham et al., 2012).

Parallel to academic advancement, socio-emotional outcomes constitute a critical dimension of these interventions. Multifaceted school–university programs have been shown to strengthen students’ self-efficacy and reconstruct their academic identity (Casey et al., 2011). Such initiatives provide not only cognitive but also emotional support to gifted learners. E-mentoring programs, for example, have assisted students in balancing academic and athletic workloads, improving time management, and coping with setbacks thereby enhancing self-regulation and resilience (Lamb & Aldous, 2014).

However, the ‘gifted’ label does not always yield positive outcomes. Research indicates that the label can cause emotional stress, creating a conflict where students feel torn between the pride of recognition and the fear of social exclusion (Freeman, 2013; Graham et al., 2012; Lamb & Lane, 2013). Therefore, educators must handle the identification process with care to mitigate these negative psychological effects. Cross-cultural studies further highlight that gifted students frequently face jealousy, social isolation, and stigmatization within peer groups. As a coping mechanism, many resort to concealing their abilities or conforming to peer expectations (Netz & Lefstein, 2016). Such emotional strategies, though adaptive in the short term, can undermine self-esteem and belonging, indicating that educational interventions must address social and emotional well-being alongside cognitive development.

Inequality and family support also emerge as defining variables in socio-emotional outcomes. Research by Koshy et al. (2013) revealed that families living in relative poverty face substantial barriers in accessing identification and enrichment programs for their gifted children. In contexts where school guidance capacity is limited, these barriers impede both academic progress and psychological resilience. Similarly, Dimitriadis et al. (2021) found that teachers’ limited knowledge and confidence in working with twice exceptional (2e) learners negatively affect both their academic engagement and emotional participation.

Taken together, these findings indicate that the impact of gifted education interventions is highly context dependent. The most successful outcomes occur when teacher expertise is strong, when programs adopt small-group or mentoring-based formats, and when family and school ecosystems provide cohesive support (Dimitriadis, 2012a; Casey et al., 2011). Theoretical grounding, equitable access, and systematic guidance emerge as essential components for achieving sustainable academic and socio-emotional development. Ultimately, the success of gifted education should not be measured solely by cognitive indicators but must encompass emotional well-being, sense of belonging, and lifelong learning motivation.

Table 6 illustrates that gifted education in England is examined through two complementary dimensions: academic and socio-emotional outcomes. Academic gains are shown to strengthen with teacher expertise, program duration, and a solid theoretical foundation, whereas socio-emotional outcomes are primarily shaped by family support, sense of belonging, and psychological resilience. The findings highlight that these two domains are not independent but function in constant interaction. Overall, the table emphasizes that effective gifted education should address not only cognitive achievement but also students’ emotional well-being and social adjustment.

### 4.5. RQ5: What Methodological Patterns and Quality Characteristics Are Observed in Gifted Education Research Conducted in England?

A comprehensive analysis of studies on gifted education in England reveals four dominant methodological trends that together illustrate the evolving nature of research in this field. The first and most prevalent tendency is the reliance on qualitative and small-sample research designs, which prioritize ecological validity over experimental control. Such studies emphasize the lived experiences of teachers, coordinators, and students within authentic school environments, often examining how policy, pedagogy, and teacher behaviour intersect in daily practice. Notably, case studies and school-based inquiries at the primary level (Dimitriadis, 2012a, 2012b), reflective policy analyses (Koshy et al., 2010b) and qualitative explorations incorporating student and parent perspectives (Lamb & Lane, 2013; Koshy et al., 2013) dominate the landscape. These works typically integrate semi-structured interviews, focus group discussions, classroom observations, and documentary analyses of institutional and policy documents, using thematic and content analysis to interpret findings. While this body of work contributes rich contextual insights, its limited generalizability and lack of standardized evaluation frameworks restrict the potential for cumulative evidence building across settings.

The second methodological pattern evident in the literature involves mixed-method and programme evaluation approaches, which combine quantitative performance indicators with qualitative reflection to assess intervention effectiveness. For example, Casey et al. (2011) implemented a longitudinal university–school partnership to support high-ability students from disadvantaged backgrounds, integrating achievement data with interview-based insights. Similarly, Dimitriadis (2012a) compared four models of educational provision: classroom differentiation, ability grouping, enrichment programs, and mentoring within a multiple case study framework. These designs enhanced internal validity through triangulation and context-sensitive interpretation. However, they were often constrained by short implementation periods, small sample sizes, and limited replicability, underscoring the need for more systematically designed and longitudinally sustained evaluations.

A third body of research consists of targeted pilot and cross-sectional studies addressing specific conceptual and policy gaps in gifted education. Dimitriadis et al. (2021), for instance, explored teachers’ knowledge of “twice exceptional” (2e) students those who combine high ability with learning or behavioural challenges and revealed major inconsistencies in teacher preparedness and policy alignment. Likewise, Cross et al. (2019) examined the social stigmatization and coping mechanisms of gifted students through cross-cultural qualitative inquiry, providing valuable insights into the socio-emotional dimensions of giftedness. These exploratory designs play an essential role in hypothesis generation and conceptual refinement, but their intentionally limited external validity and methodological heterogeneity constrain broader generalization. Longitudinal studies provide unique perspectives on the developmental trajectories of gifted individuals. Freeman’s (2013) 35-year longitudinal investigation remains a seminal contribution, demonstrating that the “gifted” label does not consistently predict adult success. Rather, sustained achievement was more closely associated with perseverance, emotional support, and family stability. Such longitudinal perspectives add temporal depth to the field and underscore the need for research frameworks that trace giftedness as an evolving life-course phenomenon rather than a static trait. Taken together, these methodological patterns depict a research field characterized by strong contextual sensitivity and ecological realism. Yet, it is also one that continues to face significant challenges in establishing causal inference, standardized assessment criteria, and longitudinal continuity. The scholarship on gifted education, while methodologically diverse and reflective, would benefit from more systematically designed comparative studies and integrated evaluation frameworks that can link educational interventions with both short-term and long-term learner outcomes.

Table 7 summarizes the predominant methodological trends observed in gifted education research conducted in England between 2010 and 2025. The majority of the studies employed qualitative and case-based approaches; in addition, mixed-method evaluations and pilot studies addressing challenges related to identification and educational provision were included. Longitudinal studies provide insights into the developmental and socio-emotional processes of gifted individuals, while policy-oriented reflective analyses critically examine the evolving structure of the field following the withdrawal of national gifted education program. Research in gifted education in England demonstrates strong contextual sensitivity and qualitative richness, indicating a tendency toward interpretive inquiry rather than experimental design. Although program evaluation and mixed-methods studies offer valuable applied insights, standardized evaluation frameworks and longitudinal data capable of linking interventions to long-term developmental outcomes are lacking in the field.

## 5. Discussion

Gifted education in England has undergone a complex and often fragmented evolution over the past two decades, shaped by shifting political priorities, ideological debates, and significant disparities in implementation across schools. The national Gifted and Talented (G&T) initiative introduced in 1999 under the Excellence in Cities (EiC) programme and discontinued in 2010 represented both the most ambitious and the most contentious effort to institutionalize gifted education within the English school system. Designed to promote social mobility and academic excellence among high-potential students, particularly those from disadvantaged backgrounds, the programme nonetheless exposed deep structural and theoretical weaknesses in practice. Following the dissolution of the National Strategies and G&T support teams in 2011, schools were left to manage gifted education independently, often without a central framework or standardized guidance. The ‘rise and fall’ of the G&T programme mirrors England’s broader political transition from centralized state mandates to localized school autonomy. Its discontinuation was not merely administrative but ideological, marking a shift away from perceived ‘elitism’ toward a more inclusive, decentralized educational model. Consequently, the field became characterized by what Koshy et al. (2010a) described as a “patchwork” of local practices driven more by pragmatic necessity than by research-based or pedagogically coherent principles (Dimitriadis, 2012a, 2012b). This situation highlights a critical tension between policy intent and classroom reality. While the shift to school autonomy aimed to allow for context-specific provision, the absence of a unified framework appears to have inadvertently replaced standardization with inconsistency.

The tension between policy discourse and educational practice was most evident in the G&T programme’s identification procedures. Schools were instructed to classify between 5% and 10% of their pupils as “gifted or talented” and maintain official registers. However, this requirement generated widespread unease among educators. Throughout the post-policy period (2010–2025), the pedagogical and ethical challenges surrounding the ‘gifted’ label have persisted, exacerbated by the withdrawal of national definitions. With the removal of the specific government quota (formerly 10%), schools have struggled to define high ability, often equating it with high attainment due to a lack of theoretical coherence. In the absence of centralized guidance, identification practices have increasingly relied on narrow performance metrics, such as statutory assessment data, rather than recognizing latent potential. This continued reliance on measurable academic success has risked systematically disadvantaging students from culturally diverse or socioeconomically marginalized backgrounds, as noted in research from the early post-policy years (Koshy & Pinheiro-Torres, 2013).

Furthermore, the legacy of early identification practices has remained a contentious issue. While the formal requirement to identify students at age six was removed, many schools lacked alternative frameworks to replace it. Freeman’s (2013) longitudinal analysis, published during this period, highlighted that rigid labeling can lead to long-term psychological consequences, including perfectionism and anxiety. These findings suggest that the school-based practices evolving after 2010 often failed to capture the dynamic nature of giftedness described in holistic models, such as Renzulli’s (1978) Three-Ring Conception or Gagné’s (2004) Differentiated Model.

Crucially, the deficit in teacher expertise has deepened since the dismantling of the National Strategies in 2011. Without the specific scaffolding previously provided by local coordinators, general classroom teachers have been left with limited training in diagnosis or differentiation. Consequently, in the 2010–2025 landscape, professional development has become fragmented and rare, hindering the implementation of research-informed provision. Dimitriadis et al. (2021) found that teachers who had received basic training in gifted education showed no significant improvement in confidence or understanding particularly regarding twice-exceptional (2e) learners compared to those without any training. This pattern illustrates that policy directives alone are insufficient without sustained investment in teacher education. Moreover, inconsistencies in instructional models mirrored inconsistencies in identification. Dimitriadis’ (2012a, 2012b) comparative case studies demonstrated that approaches involving subject specialists, such as mentoring or pull-out groups, yielded more positive outcomes than mixed-ability classroom groupings, particularly in mathematics, where focused attention and advanced content facilitated higher cognitive engagement and motivation.

Mathematics education, in fact, has remained a central focus in English gifted education research. Teachers often equated rapid task completion with intellectual giftedness, neglecting deeper conceptual reasoning. This misconception narrowed the understanding of giftedness and marginalized students who demonstrated creative or divergent thinking. Particularly concerning were twice-exceptional learners students possessing both high cognitive potential and learning difficulties such as dyslexia, ADHD, or ASD who were frequently overlooked due to teachers’ limited diagnostic awareness and inadequate preparation (Dimitriadis, 2016). These findings highlight that effective gifted education requires not only enriched curricula but also pedagogical depth grounded in developmental psychology, special education, and cognitive diversity.

Beyond structural and pedagogical challenges, research has increasingly emphasized the social and emotional dimensions of giftedness. Freeman’s (2013) longitudinal findings showed that gifted individuals often carry the emotional burden of high expectations, social isolation, and perfectionist pressures well into adulthood. Cross et al. (2019), in a cross-cultural study involving England, the United States, Ireland, France, and South Korea, demonstrated that such experiences are not confined to any single national context. Gifted students frequently reported jealousy, exclusion, and the need to conceal their abilities to achieve social acceptance phenomena consistent with the “stigma of giftedness” framework. In the English context, these issues are intensified by an inclusive educational ethos that, while striving for equity, may inadvertently fail to address the specific developmental needs of gifted learners.

Subject-specific studies further illuminate these psychosocial dynamics. Lamb and Aldous (2014) observed that gifted students in physical education struggled to balance academic demands with athletic commitments and felt that their sporting achievements were undervalued within the academic hierarchy. Teachers, in turn, often regarded athletic talent as extracurricular rather than integral to learning. Similarly, Graham et al. (2012) found that students identified as highly able in modern foreign languages or sports experienced conflicting pressures from teachers and peers, resulting in stress, reduced motivation, and uncertainty about their gifted identity. Collectively, these findings indicate that the English education system continues to privilege traditional academic domains while marginalizing artistic, physical, and linguistic talents thus limiting a holistic understanding of giftedness as a multifaceted construct.

Socioeconomic disparities further compound these challenges. Koshy et al. (2013) reported that parents from low-income backgrounds often lack the resources, confidence, and institutional knowledge required to advocate effectively for their gifted children. Many described feelings of exclusion from schools and uncertainty in navigating educational pathways. Complementary evidence from Casey et al.’s (2011) university-based intervention programme for gifted students in urban, disadvantaged contexts demonstrated that sustained, multi-faceted support can foster self-confidence, academic motivation, and higher-education aspirations. Nonetheless, persistent structural challenges such as irregular attendance and foundational skill gaps limited the programme’s scalability and overall impact.

Taken together, these findings suggest that gifted education in England remains caught between aspiration and fragmentation. The national commitment to equity has often been achieved at the expense of conceptual clarity and continuity, leaving schools to operate within disjointed and inconsistent frameworks. The dismantling of the G&T programme revealed deeper systemic vulnerabilities in policy coherence, teacher training, and theoretical grounding. Moreover, the persistent neglect of social emotional development and the enduring effects of socioeconomic inequality continue to constrain the holistic growth of gifted learners. The limited volume of recent research identified in this review highlights a noticeable reduction in scholarly focus following the withdrawal of national policy support. This trend is particularly evident in the scarcity of studies addressing specific domains such as mathematics provision, twice-exceptionality, and student voice, indicating a stagnation of empirical inquiry in these critical areas over the last decade. Moving forward, it is recommended that England’s gifted education system should adopt a more integrated and sustainable model one that unites cognitive challenge with emotional support, theory with practice, and equity with excellence. Genuine progress will depend not merely on reinstating national initiatives but on embedding long-term, research-based strategies that align identification, pedagogy, and psychosocial development within a coherent and inclusive framework capable of nurturing both potential and personhood.

## 6. Limitations

Several limitations of this systematic review should be acknowledged. First, the number of studies included in the review was limited, reflecting both the specific nature of the research focus and the development of gifted education research in England following the discontinuation of the national Gifted and Talented programme.

Second, this review was restricted to peer-reviewed empirical studies published in academic journals. While this criterion ensured methodological rigor and quality, it may have excluded relevant grey literature, policy reports, and practitioner-focused evaluations that could provide additional insights into gifted education practices at the school level.

## 7. Conclusions

This systematic review synthesized fifteen peer-reviewed studies published between 2010 and 2025 to examine how gifted education in England has evolved in the absence of a national policy framework following the discontinuation of the Gifted and Talented (G&T) programme in 2010. As the first substantive review of this period, the findings are significant because they reveal a foundational concentration in the literature on the deficiencies in the identification and provision for gifted students. The main areas of focus, the gaps between policy aims and educational practice, teacher capacity, and the social and emotional dimensions of giftedness, collectively demonstrate the depth of the systemic challenges in these fields.

When studies are examined thematically, the most frequently investigated topic (f = 8) is teacher capacity. With the individualization of gifted education following policy shifts, the competence and sufficiency of teachers have emerged as a critical barrier. Studies consistently report that educators receive insufficient training in both the identification and provision for gifted students, particularly concerning twice-exceptional (2e) learners whose dual profiles require specialized knowledge (Dimitriadis et al., 2021). Professional development opportunities are often characterized by their short-term duration and lack of depth, leaving educators reliant on intuition rather than established frameworks. Evidence from mathematics education further underscores this challenge: while specialist-led mentoring programs yielded strong academic gains, these crucial approaches were not widely available due to resource constraints and a lack of systemic support (Dimitriadis, 2012b). Ultimately, this situation has led to confusion among teachers regarding how to effectively identify and educate their gifted students.

The second most frequently investigated topic (f = 7) when examining the studies thematically relates to the social and emotional dimensions of giftedness. The reviewed literature consistently demonstrates that academic success does not shield gifted students from emotional vulnerability. Longitudinal and cross-cultural findings prove that gifted learners frequently experience heightened pressure, perfectionism, social isolation, and peer stigmatization (Freeman, 2013; Cross et al., 2019). Furthermore, research in physical education and modern foreign languages indicates that non-traditional domains of ability are often trivialized, causing gifted students in these fields to struggle with identity formation, motivation, and belonging (Lamb & Aldous, 2014; Graham et al., 2012). These outcomes reinforce the critical need for socio-emotional learning to be integrated into gifted education policy and practice.

When studies are examined thematically, one of the most frequently investigated issues (f = 6) is the enduring gap between policy aims and educational practice. Although the Gifted and Talented (G&T) initiative aimed to widen access to advanced learning, its implementation consistently lacked a theoretical foundation and cohesive guidance. Research demonstrates that many educators operated without a clear understanding of national standards and relied heavily on attainment-based indicators, which led to arbitrary and often inequitable identification processes (Koshy et al., 2010a; Dimitriadis, 2012a). The collapse of the G&T project led to the fragmentation of the general framework for gifted education, which was subsequently entrusted to the individual discretion of schools.

Our study reveals an enduring, persistent gap between policy aims and classroom implementation in the education of gifted students in England. This disconnect may be attributed to the transition of responsibility to schools, where access to specialized professional development has been variable. As the literature suggests, in the absence of a centralized framework, implementation appears to depend increasingly on individual teacher initiative and local priorities, leading to diverse practices across the region. Identification processes continue to prioritize narrow, test-based assessments and subjective teacher judgment, thereby neglecting creativity and diversity. This situation consequently deepens opportunity inequity for disadvantaged and twice-exceptional (2e) students. Our most critical findings indicate that insufficient teacher capacity and a lack of support in teacher training necessitate a stronger focus on the high social pressure, isolation, and emotional vulnerability experienced by gifted students, even when academic success is achieved. Based on the synthesis of these findings, three key implications for policy and practice are proposed. First, to address the current fragmentation identified in the literature, a more cohesive yet flexible guidance framework would be beneficial to support schools in identifying potential beyond narrow metrics. Second, given the reported gaps in professional competence, integrating specific modules on gifted education including twice-exceptionality into initial teacher training warrants consideration to ensure sustainable practice. Third, the evidence regarding socio-emotional vulnerability necessitates that psychosocial support be conceptualized as an integral component of talent development frameworks, rather than a peripheral adjunct. Finally, there remains a pressing need for contemporary empirical research to revitalize the evidence base.

## Figures and Tables

**Table 1:** Keyword Combinations Used in the Literature Search.

Search String	Target Context
Gifted AND England	Studies focusing on gifted education in England
Gifted AND UK	Studies covering the United Kingdom context
Gifted AND High Ability	Capturing studies using “high ability” as an alternative to “gifted”
Gifted AND England and Wales	Research addressing both England and Wales

**Table 2:** Inclusion and Exclusion Criteria for Article Selection.

Criteria	Inclusion	Exclusion
Time Frame	Studies published between 2010–2025	Studies published before 2010 or after 2025
Language	English	Non-English publications
Publication Type	Peer-reviewed journal articles	Unpublished research, conference abstracts, book chapters, editorials, letters, doctoral or master’s theses
Geographical Focus	Focus on England (including England and Wales)	Studies exclusively on gifted education in other countries
Discipline Areas	Education, Educational Science, Psychology, Social Sciences, Humanities	Studies outside these fields (e.g., STEM-only technical reports without educational context)
Accessibility	Full-text available	Articles without full-text access
Methodology	Studies with clear methodological description	Studies with unclear or missing methodology
Content Focus	Studies addressing gifted education, policies, practices, identification, or student support	Studies focusing solely on non-gifted education practices or tangential issues unrelated to gifted education in England

**Table 3:** Methodological Characteristics of Gifted Education Research in England.

Title of the Study	Author/s	Participant	Design	Data Collection
Twice-Exceptional Students of Mathematics in England: What Do the Teachers Know?	Dimitriadis et al. (2021)	Teachers	Quantitative	Survey with Likert type and open-ended questions
Gifted Programs Cannot Be Successful Without Gifted Research and Theory: Evidence From Practice With Gifted Students of Mathematics	Dimitriadis (2016)	Teachers and their identified gifted students	Mixed method	While case study, interview, observation, documentary evidence and thematic analysis were used for the qualitative aspect, a survey was used for the quantitative aspect.
The landscape of gifted and talented education in England and Wales: how are teachers implementing policy?	Koshy et al. (2010b)	School coordinators and teachers	Mixed method	A quantitative survey was applied to school coordinators, and qualitative interviews were conducted with teachers.
Are we being de-gifted Miss?’ Primary School Gifted and Talented Co-ordinators’ responses to the Gifted and Talented Education Policy in England.	Koshy and Pinheiro-Torres (2013)	Education coordinators	Mixed method	While a survey was used for the quantitative method, an interview was conducted for the qualitative method.
How Are Schools in England Addressing the Needs of Mathematically Gifted Children in Primary Classrooms? A Review of Practice	Dimitriadis (2012a)	Gifted students and their teachers	Qualitative	Case studies using interviews, observations, and document analysis
Provision for mathematically gifted children in primary schools: an investigation of four different methods of organisational provision	Dimitriadis (2012b)	Gifted students and their teachers	Qualitative	Case studies with observations, interviews, and document analysis
Exploring the views of parents of high ability children living in relative poverty	Koshy et al. (2013)	Parents of gifted children	Qualitative	Semi-structured interviews
A Cross-Cultural Study of the Social Experience of Giftedness	Cross et al. (2019)	Gifted students from diverse ethnic backgrounds	Qualitative	Semi-structured interviews
The role of E-Mentoring in distinguishing pedagogic experiences of gifted and talented pupils in physical education	Lamb and Aldous (2014)	Gifted students and their mentors from sports sciences	Mixed methods	Surveys for quantitative data; focus group discussions and email correspondences for qualitative insights
The long-term effects of families and educational provision on gifted children	Freeman (2013)	Gifted students	Mixed methods	An intelligence test was used for the quantitative aspects and a semi-structured interview was used for the qualitative method
Pupil voice on being gifted and talented in physical education: ‘They think it’s just, like, a weekend sort of thing’	Lamb and Lane (2013)	Gifted students	Qualitative	Semi-structured interviews
Learners’ perceptions of being identified as very able: Insights from Modern Foreign Languages and Physical Education	Graham et al. (2012)	Gifted students	Mixed methods	Surveys (quantitative), semi-structured interviews (qualitative)
A cross-cultural analysis of disagreements in classroom discourse: Comparative case studies from England, the United States, and Israel	Netz and Lefstein (2016)	Typically developing and gifted students	Qualitative	Interviews and observations
Teachers’ responses to the gifted and talented policy in the UK: a review of the landscape	Koshy et al. (2010a)	Primary school teachers living in England and Wales.	Quantitative	A survey was used. The survey included open-ended and closed-ended questions.
Opportunities and Challenges of Working with Gifted and Talented Students in an Urban Context: A University-Based Intervention Program	Casey et al. (2011)	Disadvantaged ethnically diverse gifted students	Mixed	Quantitative data attendance charts of students participating in the program National academic tests Parental opinions and field notes for qualitative data

**Table 4:** Thematic Clusters of Gifted Education Research in England.

Theme	Frequency	Interpretation
Teacher Capacity	8	Examines the knowledge, skills, and confidence levels of teachers regarding the identification of gifted students, the design of differentiated instruction, and the creation of enriched learning environments.
Policy and Pedagogy	6	Covers the dynamics and consequences of the shift from centralized mandates to localized implementation of the education framework in England, particularly after the discontinuation of the Gifted and Talented (G&T) program.
Student Voice	4	Highlights gifted students’ own feelings, perceptions, and perspectives on their educational experiences, labelling, and academic support mechanisms.
Equity and Family Context	3	Socioeconomic inequalities shape access; parental support and aspirations critical.
Well-Being and Social Experience	7	Gifted students face stigma, labelling, and social-emotional challenges.
Mathematics Provision	3	Subject-specific emphasis highlights challenges in mathematics education.

**Table 5:** Gifted Student Identification Models: Approaches, Characteristics, and Limitations.

Model/Framework Type	Key Literature	Identification Approach	Key Findings/Features	Limitations and Challenges
1. Policy-Based Frameworks and National Standards	Koshy et al. (2010b); Koshy and Pinheiro-Torres (2013)	Centralized G&T Programme (1999–2011); national IQS and CQS standards; schools required to identify top 5–10% as “gifted.”	Created first unified national structure for gifted identification; increased visibility of high-ability learners; promoted accountability and institutional coordination.	Inconsistent implementation across regions; overreliance on test-based criteria; conceptual confusion among teachers; equity and labelling issues after programme termination.
2. Teacher-Led and School-Based Models	Dimitriadis (2012a, 2012b); Casey et al. (2011)	Teacher observation, classroom performance, creative productivity, contextual judgment; organizational models (differentiation, ability grouping, enrichment, mentoring).	Greater flexibility and contextual relevance; mentoring and small-group work proved most effective for motivation and academic progress, especially when led by subject experts.	Variation in teacher competence; unequal resource distribution; inconsistent implementation; dependence on school context.
3. Theoretical and Cognitive Approaches	Dimitriadis (2016); Dimitriadis et al. (2021)	Incorporation of Renzulli’s Three-Ring Model, Gagné’s DMGT, and Bloom’s taxonomy for higher-order thinking and metacognitive assessment.	Shift from static IQ to dynamic performance-based evaluation; emphasizes creativity, problem-solving, and deep conceptual understanding.	Lack of consistent theoretical grounding; limited teacher familiarity with cognitive models; insufficient application to twice-exceptional (2e) profiles.
4. Contextual and Socio-Emotional Approaches	Koshy et al. (2013); Freeman (2013); Lamb and Aldous (2014); Graham et al. (2012)	Emphasis on socio-emotional well-being, environmental influences, and family context; focus on inclusion and cultural responsiveness.	Recognition of giftedness as contextually and emotionally situated; promotes holistic understanding integrating academic and affective dimensions.	Persistent inequities in access; lack of teacher preparation for socio-emotional dimensions; limited systemic integration into policy and practice.

**Table 6:** Conceptual Framework of Academic and Socio-Emotional Outcomes in Gifted Education Interventions (England Context).

Outcome Domain	Key Indicators	Supporting Evidence (Representative Studies)	Moderating/ Mediating Factors	Implications
Academic Outcomes	Achievement gains, conceptual depth, motivation, higher education aspirations	Casey et al. (2011); Dimitriadis (2012a, 2012b, 2016)	Teacher expertise, program duration, theoretical grounding	Sustained academic growth requires theory-based, teacher-led models emphasizing conceptual engagement and continuity.
Learning Motivation and Self-Regulation	Intrinsic motivation, task persistence, academic self-confidence	Lamb and Aldous (2014); Casey et al. (2011)	Mentorship, digital or hybrid support systems	E-mentoring and enrichment programs enhance self-regulation and learning autonomy among gifted students.
Socio-Emotional Well-Being	Self-efficacy, resilience, emotional stability, belonging	Casey et al. (2011); Freeman (2013); Cross et al. (2019)	Family and school support, peer acceptance	Programs integrating emotional support and community engagement improve both well-being and retention.
Identity and Labeling Effects	Gifted identity, labeling pressure, social integration	Lamb and Lane (2013); Graham et al. (2012); Freeman (2013)	Policy framing, cultural attitudes toward giftedness	Labelling can both affirm and alienate; emotional scaffolding and inclusive classroom culture mitigate negative effects.
Equity and Access	Participation among disadvantaged groups, parental involvement, cultural inclusion	Koshy et al. (2013); Dimitriadis et al. (2021)	Socio-economic context, teacher awareness, resource distribution	Equity-focused outreach and teacher training are essential to address systemic disparities.

**Table 7:** Methodological Patterns and Quality Characteristics in Gifted Education Research in England.

Category	Description of Methodological Pattern	Representative Studies
1. Qualitative and Case Study Designs	Emphasis on school-based qualitative inquiry, small samples, and contextual depth; focuses on lived experiences of teachers, students, and coordinators.	Koshy et al. (2010b); Koshy and Pinheiro-Torres (2013); Lamb and Lane (2013)
2. Mixed-Methods and Program Evaluation	Integration of quantitative and qualitative data to evaluate intervention effectiveness and program design (e.g., longitudinal mentoring or enrichment programs).	Casey et al. (2011); Dimitriadis (2012a, 2012b)
3. Pilot Exploratory Research	Small-scale studies investigating teacher awareness, identification gaps, and dual exceptionality (2e learners).	Dimitriadis et al. (2021)
4. Longitudinal and Developmental Studies	Long-term tracking of gifted individuals’ academic and emotional trajectories; focus on social outcomes and labelling effects.	Freeman (2013)
5. Policy-Linked Reflective Analyses	Examination of educational policy evolution and implementation in G&T programs; reflective and critical approach.	Koshy et al. (2010b)

## Data Availability

No new data were created or analyzed in this study. Data sharing is not applicable to this article.

## Supplementary Materials

The following supporting information can be downloaded at: https://www.mdpi.com/article/10.3390/jintelligence14030034/s1, Table S1: PRISMA 2020 Checklist.
