# Supplementary material for: Gifted but Misunderstood? An Interpretive Systematic Review of Gifted Education Policy, Practice, and Socio-Emotional Experience in England
Source: J Intell. 2026 Feb 24;14(3):34. doi: 10.3390/jintelligence14030034 (PMC13028215; doi:10.3390/jintelligence14030034)
Supplement: Supplementary file 1 [file jintelligence-14-00034-s001.zip › jintelligence-4111718-supplementary.pdf]

PRISMA 2020 Checklist

Section and Topic	Item #	Checklist item	Location where item is reported
**TITLE**			
Title	1	Identify the report as a systematic review.	Title page: *"Gifted but Misunderstood? An Interpretive Systematic Review of Gifted Education Policy, Practice, and Socio-Emotional Experience in England"* (Title explicitly states *Systematic Review*)
**ABSTRACT**			
Abstract	2	See the PRISMA 2020 for Abstracts checklist.	Abstract section, where objectives, data sources (WoS, Scopus, Google Scholar), time frame (2010–2025), number of included studies (n=15), methodology (systematic mapping review), and key findings are clearly stated
**INTRODUCTION**			
Rationale	3	Describe the rationale for the review in the context of existing knowledge.	Introduction, paragraphs discussing the withdrawal of the G&T policy in 2010 and the resulting research gap
Objectives	4	Provide an explicit statement of the objective(s) or question(s) the review addresses.	Introduction, explicit research questions RQ1–RQ5 listed at the end of the Introduction section
**METHODS**			
Eligibility criteria	5	Specify the inclusion and exclusion criteria for the review and how studies were grouped for the syntheses.	Table 2: *Inclusion and Exclusion Criteria for Article Selection* (time frame, language, publication type, geography, discipline, methodology)
Information sources	6	Specify all databases, registers, websites, organisations, reference lists and other sources searched or consulted to identify studies. Specify the date when each source was last searched or consulted.	Methodology section 3.1; Databases: Web of Science, Scopus, Google Scholar, Last search update: November 2025
Search strategy	7	Present the full search strategies for all databases, registers and websites, including any filters and limits used.	Table 1: Keyword combinations Search strings explicitly listed (e.g., *Gifted AND England*, *Gifted AND UK*)
Selection process	8	Specify the methods used to decide whether a study met the inclusion criteria of the review, including how many reviewers screened each record and each report retrieved, whether they worked independently, and if applicable, details of automation tools used in the process.	Section 3.1 ; Two authors independently screened titles and abstracts; full texts reviewed collaboratively; disagreements resolved by discussion; no automation tools used
Data collection process	9	Specify the methods used to collect data from reports, including how many reviewers collected data from each report, whether they worked independently, any processes for obtaining or confirming data from study investigators, and if applicable, details of automation tools used in the process.	Section 3.2 Data Extraction; Manual coding; two reviewers independently extracted data; consensus-based resolution of disagreements
Data items	10a	List and define all outcomes for which data were sought. Specify whether all results that were compatible with each outcome domain in each study were sought (e.g. for all measures, time points, analyses), and if not, the methods used to decide which results to collect.	Section 3.2; Outcomes include academic outcomes, socio-emotional outcomes, identification models, methodological characteristics
	10b	List and define all other variables for which data were sought (e.g. participant and intervention characteristics, funding sources). Describe any assumptions made about any missing or unclear information.	Section 3.2; Variables: publication year, methodology, participant group, identification framework, educational outcomes
Study risk of bias assessment	11	Specify the methods used to assess risk of bias in the included studies, including details of the tool(s) used, how many reviewers assessed each study and whether they worked independently, and if applicable, details of automation tools used in the process.	Section 3.1 and 3.2; Stated explicitly that no formal risk of bias assessment was conducted due to systematic mapping review design

Section and Topic	Item #	Checklist item	Location where item is reported
Effect measures	12	Specify for each outcome the effect measure(s) (e.g. risk ratio, mean difference) used in the synthesis or presentation of results.	Section 4 (Results); No quantitative effect measures reported; meta-analysis not conducted due to heterogeneity
Synthesis methods	13a	Describe the processes used to decide which studies were eligible for each synthesis (e.g. tabulating the study intervention characteristics and comparing against the planned groups for each synthesis (item #5)).	Section 3.3 Analysis; Descriptive statistics + narrative synthesis + thematic mapping, No meta-analysis, no heterogeneity or sensitivity analyses performed
	13b	Describe any methods required to prepare the data for presentation or synthesis, such as handling of missing summary statistics, or data conversions.	Section 3.3 Analysis; Descriptive statistics + narrative synthesis + thematic mapping, No meta-analysis, no heterogeneity or sensitivity analyses performed
	13c	Describe any methods used to tabulate or visually display results of individual studies and syntheses.	Section 3.3 Analysis; Descriptive statistics + narrative synthesis + thematic mapping, No meta-analysis, no heterogeneity or sensitivity analyses performed
	13d	Describe any methods used to synthesize results and provide a rationale for the choice(s). If meta-analysis was performed, describe the model(s), method(s) to identify the presence and extent of statistical heterogeneity, and software package(s) used.	Section 3.3 Analysis; Descriptive statistics + narrative synthesis + thematic mapping, No meta-analysis, no heterogeneity or sensitivity analyses performed
	13e	Describe any methods used to explore possible causes of heterogeneity among study results (e.g. subgroup analysis, meta-regression).	Section 3.3 Analysis; Descriptive statistics + narrative synthesis + thematic mapping, No meta-analysis, no heterogeneity or sensitivity analyses performed
	13f	Describe any sensitivity analyses conducted to assess robustness of the synthesized results.	Section 3.3 Analysis; Descriptive statistics + narrative synthesis + thematic mapping, No meta-analysis, no heterogeneity or sensitivity analyses performed
Reporting bias assessment	14	Describe any methods used to assess risk of bias due to missing results in a synthesis (arising from reporting biases).	Explicit statement: no formal reporting bias assessment conducted; mitigation via multiple databases and clear criteria
Certainty assessment	15	Describe any methods used to assess certainty (or confidence) in the body of evidence for an outcome.	Section 3.2; Explicitly stated that no certainty assessment was conducted due to mapping review focus
**RESULTS**			
Study selection	16a	Describe the results of the search and selection process, from the number of records identified in the search to the number of studies included in the review, ideally using a flow diagram.	Figure 1 (PRISMA flow diagram) and Results section describing numbers screened and included (n=15)
	16b	Cite studies that might appear to meet the inclusion criteria, but which were excluded, and explain why they were excluded.	Inclusion/exclusion criteria described; reasons for exclusion summarized in Figure 1 and Table 2
Study characteristics	17	Cite each included study and present its characteristics.	Table 3: Methodological Characteristics of Included Studies
Risk of bias in studies	18	Present assessments of risk of bias for each included study.	Not assessed (systematic mapping review); explicitly stated in Methods and Limitations
Results of individual studies	19	For all outcomes, present, for each study: (a) summary statistics for each group (where appropriate) and (b) an effect estimate and its precision (e.g. confidence/credible interval), ideally using structured tables or plots.	Results section; narrative synthesis supported by Tables 3–8
Results of syntheses	20a	For each synthesis, briefly summarise the characteristics and risk of bias among contributing studies.	Section 4 Results; thematic synthesis (RQ1–RQ5); no statistical synthesis performed

PRISMA 2020 Checklist

Section and Topic	Item #	Checklist item	Location where item is reported
	20b	Present results of all statistical syntheses conducted. If meta-analysis was done, present for each the summary estimate and its precision (e.g. confidence/credible interval) and measures of statistical heterogeneity. If comparing groups, describe the direction of the effect.	Section 4 Results; thematic synthesis (RQ1–RQ5); no statistical synthesis performed
	20c	Present results of all investigations of possible causes of heterogeneity among study results.	Section 4 Results; thematic synthesis (RQ1–RQ5); no statistical synthesis performed
	20d	Present results of all sensitivity analyses conducted to assess the robustness of the synthesized results.	Section 4 Results; thematic synthesis (RQ1–RQ5); no statistical synthesis performed
Reporting biases	21	Present assessments of risk of bias due to missing results (arising from reporting biases) for each synthesis assessed.	Not formally assessed; explicitly stated
Certainty of evidence	22	Present assessments of certainty (or confidence) in the body of evidence for each outcome assessed.	Not assessed; explicitly stated
**DISCUSSION**			
Discussion	23a	Provide a general interpretation of the results in the context of other evidence.	Section 5 Discussion (interpretation, evidence limitations, review limitations, policy/practice implications)
	23b	Discuss any limitations of the evidence included in the review.	Section 5 Discussion (interpretation, evidence limitations, review limitations, policy/practice implications)
	23c	Discuss any limitations of the review processes used.	Section 5 Discussion (interpretation, evidence limitations, review limitations, policy/practice implications)
	23d	Discuss implications of the results for practice, policy, and future research.	Section 5 Discussion (interpretation, evidence limitations, review limitations, policy/practice implications)
**OTHER INFORMATION**			
Registration and protocol	24a	Provide registration information for the review, including register name and registration number, or state that the review was not registered.	No registration; protocol not prepared (stated implicitly under limitations and methods)
	24b	Indicate where the review protocol can be accessed, or state that a protocol was not prepared.	No registration; protocol not prepared (stated implicitly under limitations and methods)
	24c	Describe and explain any amendments to information provided at registration or in the protocol.	No registration; protocol not prepared (stated implicitly under limitations and methods)
Support	25	Describe sources of financial or non-financial support for the review, and the role of the funders or sponsors in the review.	Funding section: “This research received no specific grant”
Competing interests	26	Declare any competing interests of review authors.	Conflicts of Interest section: none declared
Availability of data, code and other materials	27	Report which of the following are publicly available and where they can be found: template data collection forms; data extracted from included studies; data used for all analyses; analytic code; any other materials used in the review.	Data Availability Statement: Not applicable

From: Page MJ, McKenzie JE, Bossuyt PM, Boutron I, Hoffmann TC, Mulrow CD, et al. The PRISMA 2020 statement: an updated guideline for reporting systematic reviews. BMJ 2021;372:n71. doi: 10.1136/bmj.n71.
This work is licensed under CC BY 4.0. To view a copy of this license, visit https://creativecommons.org/licenses/by/4.0/
